# Numerical Simulation Study of Expanding Fracture of 45 Steel Cylindrical Shell under Different Detonation Pressure

**DOI:** 10.3390/ma15113980

**Published:** 2022-06-03

**Authors:** Zhenwei Huang, Xinlu Yu

**Affiliations:** 1 Key Laboratory of Impact and Safety Engineering, Ningbo University, Ministry of Education, 818 Fenghua Road, Jiangbei District, Ningbo 315211, China; hzw961020@163.com; 2 College of Science & Technology, Ningbo University, Wenwei Road 521, Ningbo 315300, China

**Keywords:** cylindrical shell fracture, 45 steel, SPH numerical simulation

## Abstract

Detonation and fragmentation of ductile cylindrical metal shells is a complicated physical phenomenon of material and structural fracture under a high strain rate and high-speed impact. In this article, the smoothed particle hydrodynamics (SPH) numerical model is adopted to study this problem. The model’s reliability is initially tested by comparing the simulation findings with experimental data, and it shows that different fracture modes of cylindrical shells can be obtained by using the same model with a unified constitutive model and failure parameters. By using this model to analyze the explosive fracture process of the cylindrical shells at various detonation pressures, it shows that when the detonation pressure decreases, the cylindrical metal shell fracture changes from a pure shear to tensile–shear mixed fracture. When the detonation pressure is above 31 GPA, a pure shear fracture appears in the shell during the loading stage of shell expansion, and the crack has an angle of 45° or 135° from the radial direction. When the pressure is reduced to 23 GPA, the fracture mode changes to tension–shear mixing, and the proportion of tensile cracks is about one-sixth of the shell fracture. With the explosion pressure reduced to 13 GPA, the proportion of tensile cracks is increased to about one-half of the shell fracture. Finally, the failure mechanism of the different fracture modes was analyzed under different detonation pressures by studying the stress and strain curves in the shells.

## 1. Introduction

In modern explosion research, the study of expanding fracture mode of cylindrical shells under high-speed impact loading has always been a hot topic [1,2,3,4,5]. A large number of researchers have observed the fragmentation process by changing variables in detonation experiments and used the recovered fragments to study the influencing factors of the fracture mode of the cylindrical shell. The detonation of a cylindrical shell is a process under a high-impact, high-strain rate. Cylindrical shell fracture is a difficult subject that involves both material and structural damage. Based on experiments and simulations, several researchers have proposed related assumptions and findings.

As early as 1943, Gurney [6] proposed an empirical formula for the velocity of cylindrical shell fragments based on the law of energy balance. The velocity relates to the mass of explosives, the mass of cylindrical shells, and the Gurney energy. In 1944, Taylor [7] observed the fracture process of the metal cylindrical shell under a certain explosion pressure through a high-speed camera, believed that the fracture of the cylindrical shell was a tensile fracture process and he proposed the Taylor criterion. However, the criterion did not consider other influencing factors, such as explosion pressure, shell dimensions, and loading method. Besides, the control mechanism is relatively simple. Based on the Taylor criterion, Hoggatt et al. [8] found another mode of expansion fracture under different explosion pressure. They thought that the fracture process of the cylindrical shell was a competitive fracture between tensile fracture and shear fracture, under high explosive pressure, the shell is prone to pure shear fracture. Through experiments and numerical simulation studies, Martineau [9] found that the cylindrical shell had an adiabatic shear phenomenon in the expansion process when the strain rate was 104 s^−1^ and the strain was about 150%, the thickness of the cylindrical shell also affected adiabatic shear. Studies on microscopic characterization of metal materials [10], metal thermodynamic properties [11], and the influence of impurities in steel materials on the properties of the material [12] have shown the metal failure process is affected by many factors. In recent years, Liang et al. [13] studied the expansion ring′s fracture by numerical simulation, and found that with low notch depth conditions, the expansion ring is prone to pure shear fracture. Tensile fracture dominated when the notch depth was higher, this result indicated that fracture mode was affected by defect factors. Under the guidance of the dynamic fragmentation theory of solids and the fragmentation theory of continuum energy, Grady [5] analyzed that the distribution of shell fragments was related to the ductility of the material itself. Hu et al. [14] and Hu et al. [15] carried out experimental research on the damage to metal cylindrical shells of 45 steel, TC4, and WTG05 tungsten alloys with different geometric dimensions driven by explosive detonation. They found that factors such as the thickness of the shell and loading detonation pressure would affect the fracture process of cylindrical shells. Generally speaking, thin-walled shells are prone to pure shear fracture under high detonation pressure loading, and fracture strain and strain rate would be relatively higher. When the explosion pressure is decreased or the shell thickness is increased, the fracture mode of the shell would change to shear–tensile mixed fracture, and the fracture strain and strain rate would be lower. Through numerical simulation research, Liu et al. [16] also found that different initiation methods of the explosion would affect the fracture of the cylindrical shell because the incident angle of the detonation wave to the inner wall of the cylindrical shell varied under different initiation methods of explosion; the pressure experienced by the inner surface of the cylindrical shell differs greatly. The results show that the peak pressure of the centerline detonation is the maximum, the single-point detonation is the second, and the plane detonation is the minimum. Arnold [4] studied the experimental results of metal cylindrical shells with different materials, wall thicknesses, and processing techniques, and proposed a calculation formula for predicting the distribution of shell fragments. Arnold found that medium-strength steel casings produced the largest fragments. In the axial direction, medium-strength steel casings produced the longest fragments. In terms of numerical simulation, Yu et al. [17] studied the pure shear fracture mechanism of TA2 cylindrical metal shells loaded with different explosives. Liu et al. [18] considered heterogeneity of the material and introduced a material shear failure constitutive model with probability, and discussed the initiation of cracks and propagation of multiple adiabatic shears in metal cylindrical shells.

However, the traditional numerical simulation method, especially the finite element method (FEM), can only analyze the deformation process, stress, and strain state of a cylindrical shell under an explosive shock wave, but by using the FEM method, various fracture modes such as pure shear fracture and the tensile–shear mixed fracture cannot be reproduced at present [17,18,19]. When using the traditional method (FEM) to analyze the fracture of a cylindrical shell, because the element produces extreme deformation and sudden change in the interface, it always appears that the failed element is deleted, which affects the fracture evolution process of a cylindrical metal shell in a subsequent calculation [20,21]. In addition, a large number of deleted elements lead to excessive loss of mass, momentum, and energy in the system. The method of smoothed particle hydrodynamics (SPH) adopted in this paper is a meshless method with Lagrangian particle configuration [22]; it avoids the mesh distortion of the Lagrangian method and the difficulty of the Euler method to capture the boundary. In the SPH method, the state of the system is described by discrete particles, and the smooth particles are used to bear their unique material properties so that they can work according to the law of the governing equation. By solving the integral expression of kernel function interpolation in the form of discrete particle weighted summation, a stable and smooth approximation is obtained. In addition, when the distance between two particles exceeds a failure threshold, the particles are not deleted when the material fractures. The conservation of mass, momentum, and energy of the system is guaranteed; thus, the SPH simulation method has great applications in the fields of ultra-high-speed impact, explosion, crack propagation, and metal forming [23,24,25]. Therefore, this article selected the smoothed particle hydrodynamics method to establish the cylindrical shell explosion model.

## 2. Experimental Phenomenon of Fracture of Metal Cylindrical Shell

**Figure 1:**
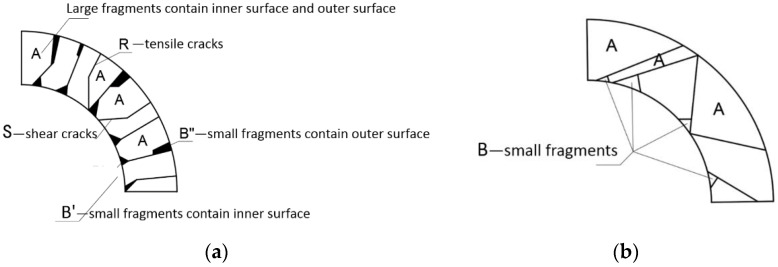
Main fragments: “A”—large fragments contain inner surface and outer surface and “B”—small fragments only contain one surface of the cylindrical shell; (**a**) tensile–shear mixed fracture mode; (**b**) pure shear fracture mode.

Many experimental studies have been carried out in China and worldwide on the fracture phenomenon and fragmentation characteristics of metal cylindrical shells under explosive loads [26,27,28]. Research shows that, for different materials and different detonation pressures, the fracture of the cylindrical shell demonstrates the following characteristics (in Figure 1): (1) Under high explosive pressure, the cylindrical shell undergoes shear fracture. When the detonation pressure decreases, it becomes a tensile–shear mixed fracture. The proportions of tensile cracks in the fracture are different; the lower the detonation pressure, the higher the proportion of tensile cracks in the fracture. (2) The shear cracks are formed by the unstable slip of the adiabatic shear belt. The direction is 45° or 135° to the radial direction. The cross-section is relatively flat (S area); the direction of the tensile cracks is along the radial direction, and the cross-section is uneven and rough (R area). (3) Cylindrical shell fragments can be divided into two types: large fragments of type A and small fragments of type B, as shown in Figure 1. Large fragments of type A include two surfaces of the cylindrical shell: the inner surface and the outer surface. Small fragments of type B only contain one surface of the cylindrical shell, according to whether the contained surface is the inner surface or the outer surface; it can be divided into B′ and B′′.

However, scholars have different understandings of the fracture process and the formation mechanism of fragments [3,29,30]. It is generally acknowledged that under low detonation pressure, the radial cracks initially occur on the outer surface under circumferential stress, and the thermoplastic shear band appears close to the inner wall, forming shear–tensile mixed fracture phenomenon. Under high detonation pressure, the inner wall undergoes thermoplastic adiabatic shear instability under compressive stress. The inward development of the radial cracks in the outer surface is inhibited and it develops along the shear direction; it shows pure shear fracture at last [6], However, Zhang et al. [31] used numerical simulation to analyze the equivalent plastic strain evolution of the cylindrical metal shell in its explosion. They believed that the fracture of the ideal cylindrical shell cannot start from the outer surface. The tensile fracture starting from the outer surface may have a geometrical relationship with the defects in the surface of the shell. Zhen et al. [32] analyzed the metallographic structure of the exploding fracture fragments of the thick-walled cylinder, and believed that under low explosive detonation pressure, the microcrack damage zone was first generated inside the wall thickness of the cylindrical shell, with the explosion pressure loading, the shell fractures. Since the zone near the inner wall of the cylindrical shell is under a state of compressive stress and the outer wall is under a state of tensile stress, the crack expands along radially outwards and expands along the shear concentration zone toward the inner surface, forming a tensile–shear mixed fracture.

Since most experimental research does not systematically design the experimental loading conditions, it is difficult to carry out a univariate comparison by verification analysis of the literature results. In China, Hu et al. [15], Tang et al. [33], and Hu et al. [34] carried out a series of comparative experimental studies on cylindrical shells and found different fracture modes, such as shear fracture and tensile–shear mixed fracture; there is also a single-rotation adiabatic shear fracture phenomenon under high detonation pressure. It can be seen that there is broad understanding of the fracture process of cylindrical shells under different explosion pressures, but the fracture mechanism is not yet clear.

In Tang′s [34] experiment, JOB-9003 and hollow RHT-901 explosives were used to carry out a series of experimental studies on the explosion and fragmentation of a 45 steel cylindrical shell. Under different detonation pressures and charging conditions, the cylindrical shells of the same material, explosion, and fragmentation had pure shear fractures and tensile–shear mixed fractures.

The 45 steel cylindrical shell shows pure shear fracture under JOB-9003 explosive loading. A cylindrical shell with 4 mm thickness shows tensile–shear mixed fracture under the hollow RHT-901 explosive loading; the proportion of tensile cracks is extremely low, so it is almost pure shear fracture; when the wall thickness is 5 mm, the proportion of tensile cracks accounts for one-third of the shell thickness [33]. The cylindrical shell size, loading conditions, and fracture phenomenon are listed in Table 1. (Relative sized δ_d_ = shell thickness h/inner radius R of cylindrical shell). The average strain and strain rate of the cylindrical shell at time t were calculated using the formula: $\varepsilon_{t}=\left(R\left(t\right)-R_{0}\right)/R_{0}$, $\dot{\varepsilon }=d\varepsilon_{t}/dt\approx V_{t}/R_{0}$. The statistical experimental data are shown in Table 1 [15,33,34].

## 3. SPH Numerical Simulation Model and Verification

### 3.1. Model Construction

**Figure 2:**
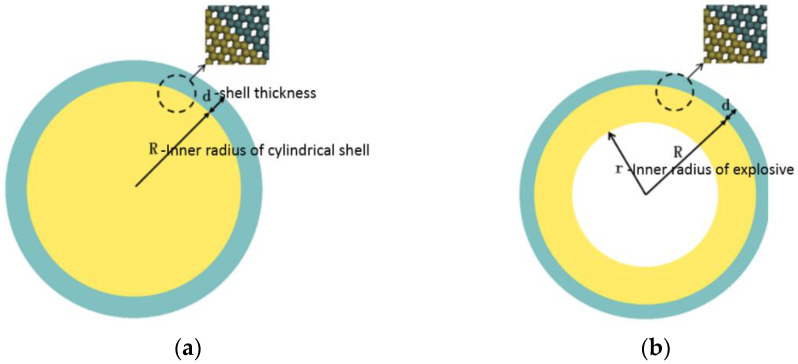
SPH numerical model: (**a**) JOB9003 packing (R = 30 mm) and (**b**) RHT-901 hollow packing (R/r = 30 mm/20 mm).

When building the SPH model, the arrangement method of the particles is very important, which affects the accuracy of the result. LS-DYNA software provides automatic polar coordinate point distribution mode and the rectangular coordinate point distribution mode, but these two methods cause local uniform particle dispersion on the contact surface of the explosive metal that are not in one-to-one correspondence. Therefore, while building the cylindrical metal shell model, it is necessary to simultaneously satisfy the uniform dispersion of particles in the explosive propagation process and the symmetry of the interface contact between the annular explosive and the metal cylinder shell. It is required that the particle arrangement and spacing of the SPH model should be kept as uniform as possible, and the interparticle symmetrical arrangement of the interface should be maintained. For this reason, the initial geometrical arrangement of particles is controlled by geometric grid cell division during modeling. Then, the method of generating particles at the grid cell center is used to ensure that the final arrangement and distribution of SPH particles are uniform and the particle pairs at the interface are symmetrically arranged, as shown in Figure 2.

**Figure 3:**
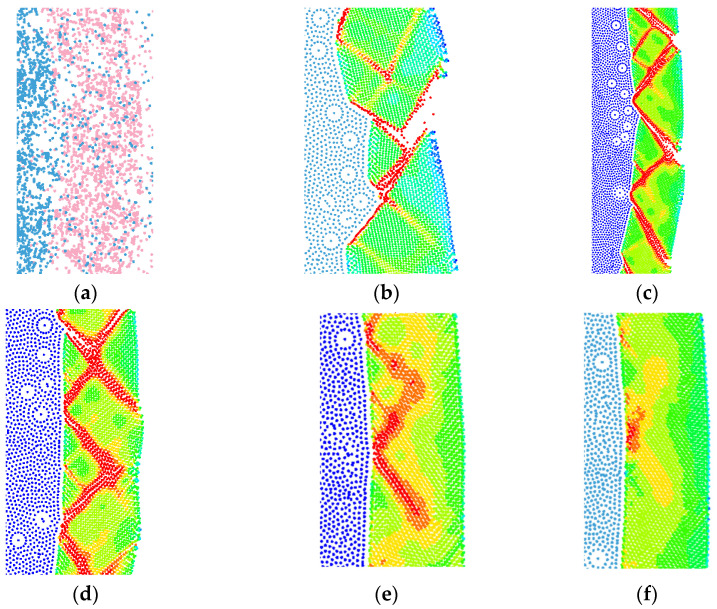
Comparison of the effects of different β values: (**a**) 14.2 μs, β = 0.8; (**b**) 14.2 μs, β = 1; (**c**) 14.2 μs, β = 2; (**d**) 14.2 μs, β = 3; (**e**) 14.2 μs, β = 7; (**f**) 14.2 μs, β = 10.

According to the literature [35,36], SPH particles have the same performance as atoms, that is, they diverge when they are close to each other and attract each other when they are far apart, thus resulting in particle aggregation during the process of calculation. Therefore, the artificial stress represented by Monaghan can effectively eliminate the tensile instability caused by SPH, so that the SPH method can maintain continuity in higher-order calculations. Monaghan artificial viscosity is controlled by parameters α and β in practical application. The β coefficient controls the penetration of interfacial particles (as shown in Figure 3). When the simulated detonation pressure is very high, it is necessary to increase the value of β to prevent nonphysical penetration, but the increase in β decreases the peak detonation pressure, increases the calculation time, and also changes the shear band development. 

To improve the accuracy and tensile stability, moving least-squares-based formulation is used. This method is suitable for large deformation models, but it requires a large computational cost. MPP simulation needs to set the maximum and minimum smooth length scale factor at 1.00 to ensure that the calculation is correct, then by adjusting a quasi-linear approximation term QL in the algorithm. To the combination of precision and stability in an extremely large deformation simulation, if QL is set too small, the model will have higher accuracy, but the stability problem is outstanding; if QL is too large, the model will inhibit instability but have lower precision. By comparison, QL = 0.01 was chosen to achieve a good compromise between stability and accuracy.

**Figure 4:**
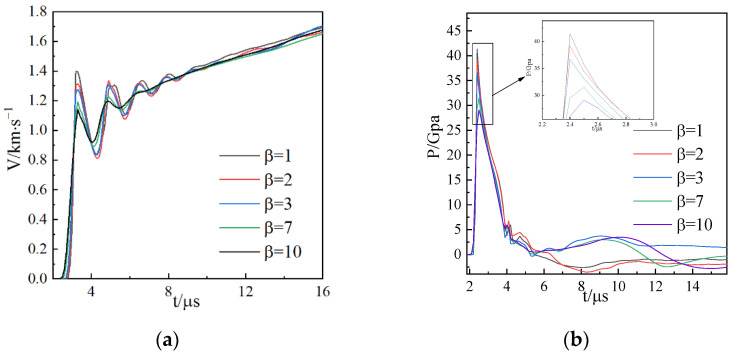
Comparison of data from different models: (**a**) velocity of the outer wall of the cylindrical shell and (**b**) internal hydrostatic pressure.

The setting of β changes the detonation pressure. With the increase in β, the value of interface load decreases (as shown in Figure 4), changing the distribution, distance, and development of the shear band in the shell thickness. When the stability of contact between particles is satisfied, no particle penetration occurs at the interface; β is required to be as small as possible to obtain a clear shear band. Therefore, β = 1 was selected as the value of its viscosity coefficient in this paper. In this way, the stability of the particles can be controlled and the failure of the shell can be accurately reflected.

The SPH distribution distance has an impact on the model accuracy and internal stress propagation. If the particle spacing distribution distance decreases, the model accuracy is improved slightly, but the calculation amount is increased greatly. Finally, the model SPH distribution distance of 50 μm is chosen; it is close to the experimental result and requires less calculation. 

### 3.2. Material Parameters of the Model

The 45 steel′s rate of change under high pressure is described by the Gruneison equation of state [37]. The plasticity is described by the Johnson–Cook constitutive equation considering strain hardening, strain rate hardening, and thermodynamic softening effects.

(1) $$\tilde{\sigma }=\left(A+B{{\bar{\sigma }}^{p}}^{n}\right)\left(1+Cln\frac{\dot{\varepsilon }}{\dot{\varepsilon_{0}}}\right)\left(1-T^{*}{}^{m}\right)$$

Adapting the experimental results of Hu [38], from the same institute as Tang, he conducted an experimental study on the strain hardening, strain rate hardening, and thermodynamic softening characteristics of 45 steel, and obtained the parameters of the Johnson–Cook constitutive equation by fitting. Since the quasi-static yield strength σ_0.2_ = 350 MPa, the fracture strength σ_b_ = 600 MPa, and the elongation of 45 steel used for the experimental cylindrical shell are 17%, the yield strength is lower than that for materials reported in the literature [38]. The strain hardening term in the Johnson–Cook constitutive equation is $\left(A+B{{\bar{\sigma }}^{p}}^{n}\right)$; the quasi-static yield strength and fracture strength properties of 45 steel used in the experiment were used for fitting and correction. The following were obtained: parameter *A* = 350 MPa, *B* = 600 MPa, *n* = 0.307. Other parameters of strain rate hardening and thermal softening are adopted directly from those reported in the literature [38], as shown in Table 2.

To facilitate the analysis, the failure model of 45 steel adopts the simplified plastic damage accumulation softening failure criterion and does not consider the influence of stress state, strain rate, or temperature on the damage. The damage degree *D* is:

(2) $$D=\{\begin{array}{ll} 0, & {\bar{\varepsilon }}^{P}<\varepsilon_{cr}\;\\ \frac{\sum \Delta {\bar{\varepsilon }}^{P}-\varepsilon_{cr}}{\varepsilon_{f}-\varepsilon_{cr}}, & {\bar{\varepsilon }}^{P}>\varepsilon_{cr} \end{array}$$

where *D* is the damage parameter,$\;\Delta {\bar{\varepsilon }}^{P}$ is the plastic variable increment of a time step, $\varepsilon_{cr}$ is the material plastic initial damage strain, and $\varepsilon_{f}$ is the material failure strain. We take $\varepsilon_{cr}\;$= 0.48 and material failure strain $\varepsilon_{f}\;$= 1.0. Once damage occurs, the material softens:

(3) $$\sigma =\tilde{\sigma }\left(1-D\right)$$

The explosive uses the JWL equation of state. The specific parameters are shown in Table 3 [39].

### 3.3. Validation of Numerical Models

**Figure 5:**
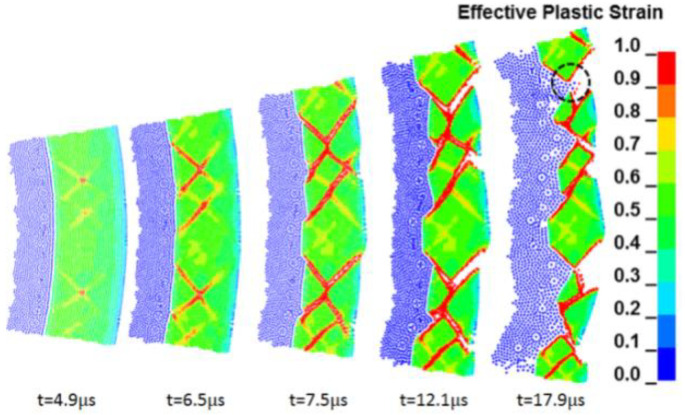
The evolution process of pure shear fracture under JOB9003 loading obtained by numerical simulation.

The experimental results of detonation and expansion of 45 steel cylindrical shell given in Table 1 are taken as the object for the SPH numerical simulation. Figure 5 is the plastic strain diagram of the fracture process for 45 steel under the loading of JOB9003. The beginning of time in the figure is defined uniformly as the moment when the detonation wave reaches the inner wall of the cylindrical shell. The SPH numerical results show that the plastic damage and fracture of the material first start in the middle of the shell thickness. The crack expands to the inner and outer surfaces along the shear direction of 45° or 135° to the radial direction. When *t* = 7.5 μs, the shear crack penetrates the shell; at that time, the shear crack surface is loaded by the internal detonation products, so the detonation products cannot leak immediately under the action of compressive stress. When *t* = 17.9 μs, the shell fragments on both sides of the crack disengaged and the detonation products leaked from the opening. The numerical simulation fracture mode is consistent with the trend of the experimental phenomenon. 

**Figure 6:**
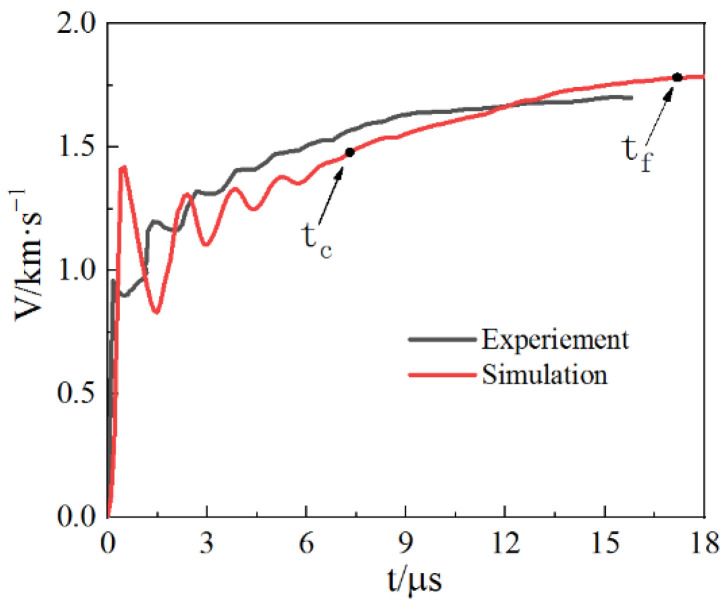
The radial expansion velocity for the outer surface of the cylindrical shell.

Figure 6 shows the comparison between the velocity–time history curve of the outer wall of the cylindrical shell obtained by SPH numerical simulation and the experimental DPS test results. They fit well; the time $t_{c}$ of visible cracks on the outer surface of the cylindrical shell and the time of detonation particle leakage $t_{f}$ are marked.

**Figure 7:**
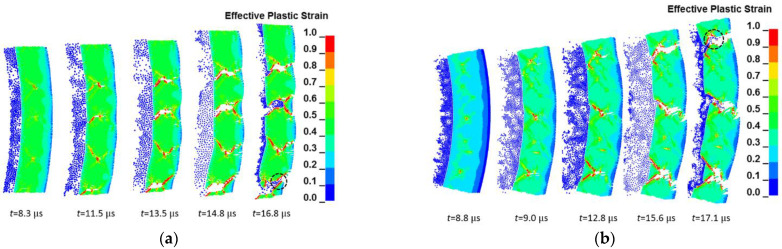
Simulation results of cylindrical shell expansion and fracture process with different R/h under a load of hollow RHT-901 explosive: (**a**) inner diameter 30 mm, shell thickness 4 mm; (**b**) inner diameter 30 mm, shell thickness 5 mm.

A numerical simulation of the fracture of 45 steel cylindrical shell with an inner diameter of 30 mm and wall thickness of 4 and 5 mm under the loading of hollow RHT-901 was carried out. The fracture model is a tensile–shear mixed fracture. Figure 7 shows the equivalent plastic strain and fracture evolution process of cylindrical shells with 4 and 5 mm thickness. It can be seen that the fracture of the cylindrical shell still starts from the middle of the wall thickness of the cylindrical shell and expands in the shear direction. In contrast to the shear evolution fracture under the action of the JOB9003 explosive, a similar “necking” phenomenon occurs at the joint of the shell when the shear crack develops close to the outer surface of the cylindrical shell. The crack direction turns to radial development. The final fracture presents a tensile–shear mixed fracture.

Among them, the shear fracture mainly occurs in the cylindrical shells with a thickness of 4 mm; tensile features can be seen locally, and the proportion of radial tensile cracks is very small, as shown in Figure 7a. The proportion of the cylindrical shell with a thickness of 5 mm has a significant increase in radial cracks, accounting for nearly one-third of the shell thickness, as shown in Figure 7b. This result is consistent with the experimental phenomenon and fracture characteristics described in the experiment [29].

Taking the detonation wave reaching the outer surface of the cylindrical shell as 0 μs, the numerical simulation results above are used in the same method as the experiment to obtain the apparent expansion strain of the cylindrical shell at the characteristic time: $\varepsilon_{t}=\left(R\left(t\right)-R_{0}\right)/R_{0}$.${\;R}_{0}$ is the initial radius of the cylindrical shell. *R*(*t*) is the expansion radius of the cylindrical shell at the characteristic time t of the detonation loading process. Finally, the initial fracture strain $\varepsilon_{c}$ at time $t_{c}$ is obtained when visible cracks are on the outer surface, together with the shell fracture strain $\varepsilon_{r}$ at time $t_{r}$ and the product leakage strain $\varepsilon_{f}$ at the explosion product leakage time $t_{f}$. The expansion strain rate $\dot{\varepsilon }$ of the cylindrical shell is approximated by the formula: $\dot{\varepsilon }={d\varepsilon }_{t}/dt\approx V_{t}/R_{0}$. $V_{t}$ is the expansion velocity when the crack penetrates the shell thickness. Under the loading of JOB9003 and hollow RHT-901 explosives, the comparison between the SPH numerical experimental results and the experiment results [29] shows that the apparent initial fracture strain $\varepsilon_{c}$ and the product leakage strain $\varepsilon_{f}$ are in good agreement, as shown in Table 4.

The results show that SPH can simulate the fracture mode of 45 steel cylindrical shell under different ways of filling and detonation pressure of JOB-9003 explosive and hollow RHT-901 explosive, which is consistent with the shear fracture, tensile–shear mixed fracture mode in experimental results. The changing trend of the fracture strain with the loading strain rate is also consistent with the experiment. The results show that the experimental end-state fragmentation phenomenon of shear and the tensile–shear mixed fracture mode under different explosive conditions can be well simulated by the SPH numerical model with the unified material constitutive and fracture model.

## 4. Fracture Results and Analysis of 45 Steel Cylindrical Shell under Different Detonation Pressure

### 4.1. Numerical Simulation Models

The abovementioned SPH simulation model (Figure 2a) was used to simulate the expansion fracture process of 45 steel cylindrical shell under four detonation pressures; the models of explosives are all fully filled SPH models with an inner diameter of 40 mm and shell thickness of h = 4 mm.

**Figure 8:**
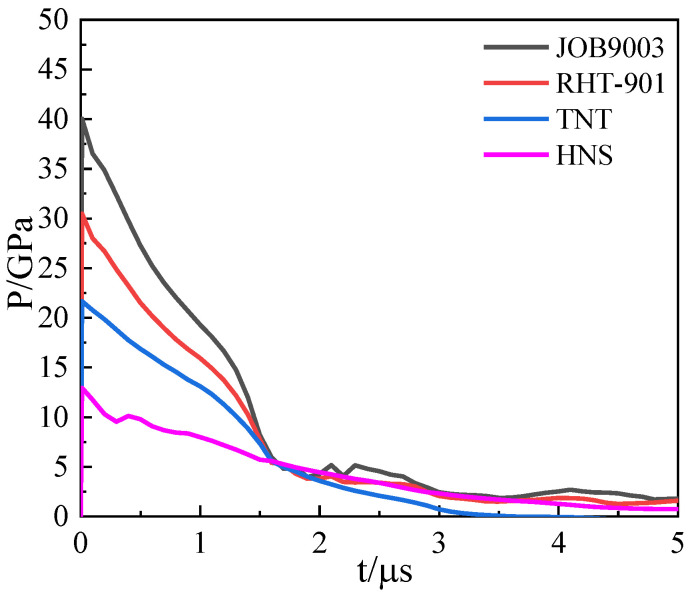
Loading history curves for the inner wall of the cylindrical shell under different explosion pressures.

The waveform is a triangular wave obtained by fitting the JWL high-pressure state equation (Figure 8). The explosive parameters are shown in Table 5.

It can be seen from the loading curve that the pulse width of the loading wave is the same, but the peaks of the loading pressures are different; the pressure peaks of the explosive detonation wave are about 40, 31, 21, and 13 GPA, and time to reach the shell inner wall was 2.2, 2.5, 2.8, and 3.8 μs. The following results take the time when the detonation wave reaches the inner wall as the starting time. Through the following results, the expansion and fracture evolution process of 45 steel under different explosive loads are analyzed.

### 4.2. Numerical Results and Analysis

**Figure 9:**
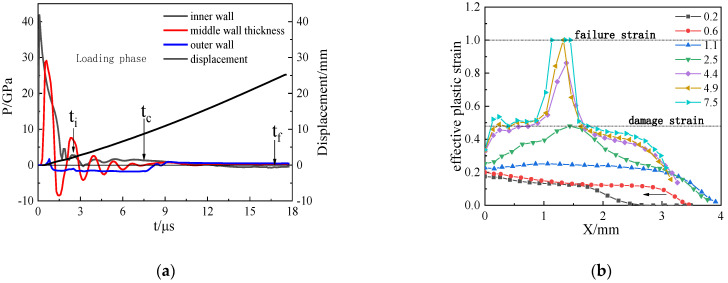

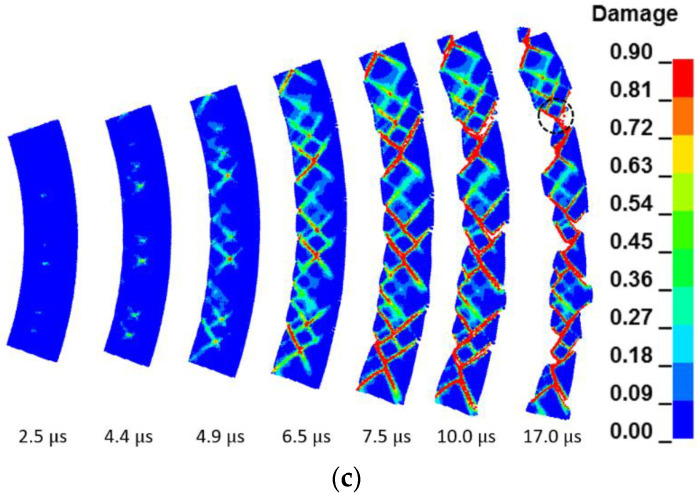
The explosive pressure, expanding deformation, and fracture for 45 steel cylinder shell under JOB9003 loading: (**a**) detonation wave propagation curves; (**b**) development of effective plastic strain in the shell thickness; (**c**) damage evolution for pure shear fracture.

Under the loading of JOB9003 explosive with high detonation pressure, the pressure propagation characteristics of the inner surface, the outer surface, and the mid-shell thickness of the 45 steel cylindrical shell with an inner diameter of 40 mm and shell thickness of 4 mm can be seen in Figure 9. Note that the inner surface is loaded with a peak value of about 40 GPA;$\;t_{i}$ represents the initial damage time, $t_{c}$ represents the cylinder shell penetration fracture time, and $t_{f}$ represents the detonation products leakage time. (1) The inner surface of the shell is loaded by detonation products and is in a state of hydrostatic stress, the outer surface is in a state of tension under the tensile stress, and the middle of the shell thickness is in a tension–compression fluctuation state. The penetration fracture of the cylindrical shell occurs in the loading stage (the inner surface of the cylindrical shell is subjected to compressive stress and regarded as the loading stage; when the pressure drops to 0, the loading stage ends), as shown in Figure 9a. (2) Before the detonation shock wave is transmitted to the outer surface of the cylindrical shell, the effective plastic strain on the inner surface accumulates the most; the effective plastic strain has a decreasing distribution from the inner wall to the outer wall. When the shock wave reaches the outer surface and is reflected, the effective plastic strain in the middle of the metal shell is the largest, and the overall distribution is convex for the secondary plastic accumulation; the strain in the middle is always the position with the largest plastic strain. It is stipulated in the previous model establishment that, when the strain accumulation reaches *D* = 0.48, the cylindrical shell material becomes damaged and softened; when the strain accumulates rapidly to *D* = 1.00, the initial failure occurs, as shown in Figure 9b; when *t* = 2.5 μs, the material damage first forms in the middle of the wall thickness. When *t* = 4.9 μs, the failure particles appear in the middle of the shell thickness and expand to the inner and outer surfaces along the 45° and 135° directions; they extend to the inner and outer surfaces of the cylindrical shell when $t_{c}\;$= 7.5 μs, forming shear cracks and penetrating the cylindrical shell. When $t_{f}$ = 17.0 μs, the detonation products leak from the opening fracture of the cylindrical shell, and the fragment fracture finally shows a pure shear shape, as indicated in Figure 9c.

**Figure 10:**
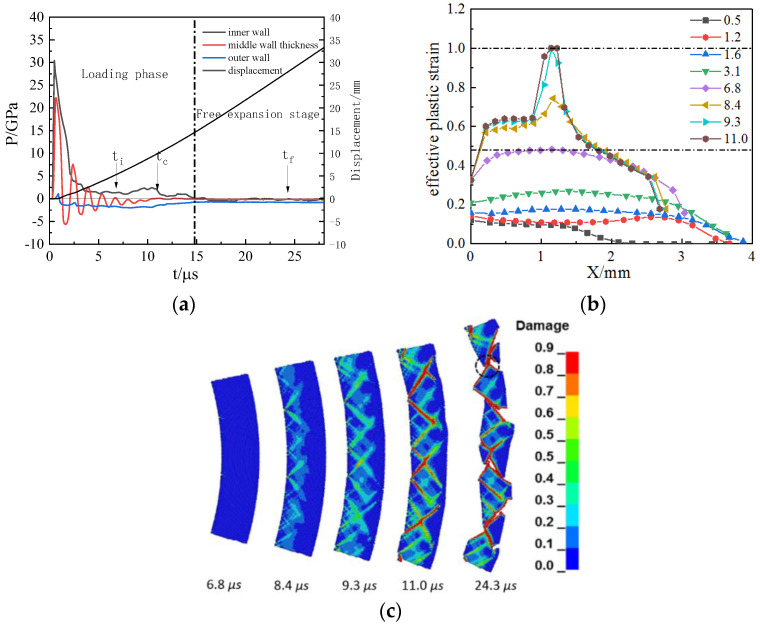
The explosive pressure, expanding deformation, and fracture for 45 steel cylinder shell under RHT−901 loading: (**a**) detonation wave propagation curves; (**b**) development of effective plastic strain in the shell thickness; (**c**) damage evolution for fracture.

The fracture process of 45 steel shell under RHT-901 loading is similar to JOB9003 loading. It shows pure shear fracture and fracture occurs in the loading stage. Under RHT−901 loading, the fracture and detonation product leakage time is later, as shown in Figure 10.

**Figure 11:**
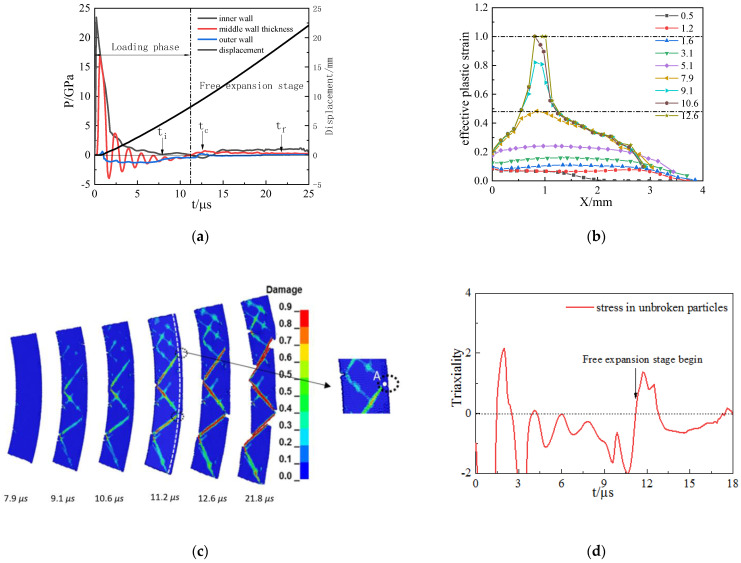
The explosive pressure, expanding deformation, and fracture for 45 steel cylinder shell under TNT loading: (**a**) detonation wave propagation curves; (**b**) development of effective plastic strain in the shell thickness; (**c**) damage evolution for fracture; (**d**) triaxial stress.

Under the loading of TNT with a lower detonation pressure, the fracture mode of the cylindrical shell shows tensile–shear mixed fracture mode. The inner surface loaded by compressive stress ends at time *t* = 11.2 μs, and then the shell enters the free expansion stage, as shown in Figure 11a. In the development of effective plastic strain in shell thickness, as shown in Figure 11b, the initial crack first forms near the inner wall; at this time, the cylindrical shell is still in the loading stage, and the crack along the 45° or 135° shear direction expands to the inner and outer surfaces. When the cylindrical shell enters the free expansion stage, *t* = 11.2 μs, the time–history curve of the triaxiality stress law at any position in the unbroken area is similar to position “A”, as shown in Figure 11d; the unbroken area of the cylindrical shell appears similar to the phenomenon of tensile “necking”—the crack development turns to the radial direction, finally becoming a tensile–shear mixed fracture. The proportion of tensile cracks is relatively small, about one-sixth, at time *t* = 12.6 μs. The cylindrical shell becomes a penetrative fracture when *t* = 21.8 μs, and detonation products leak.

**Figure 12:**
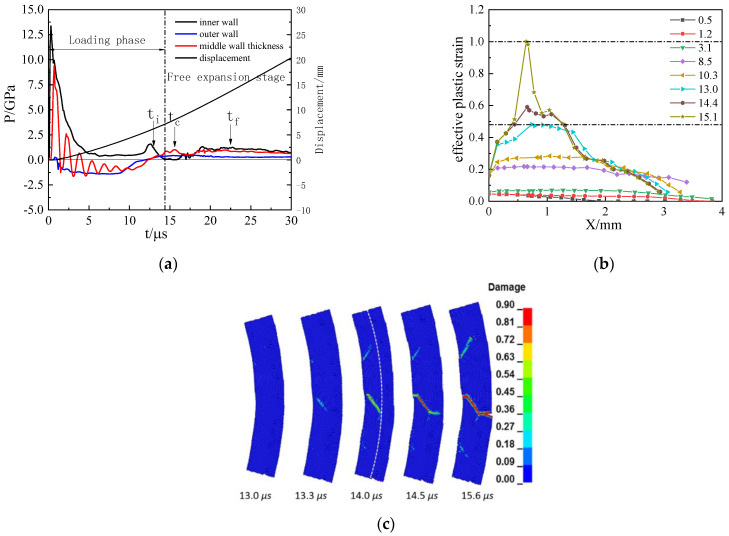
The explosive pressure, expanding deformation, and fracture for 45 steel cylinder shell under HNS loading: (**a**) detonation wave propagation curves; (**b**) development of effective plastic strain in shell thickness; (**c**) fracture damage evolution.

Under the loading of HNS with low detonation pressure, the 45 steel shell also undergoes tensile–shear mixed fracture, but tensile cracks accounted for a higher proportion in the fracture, about one-half. Shear cracks are less developed because fracture development takes less time during the loading phase, as shown in Figure 12.

## 5. Conclusions

The following conclusions were drawn from the SPH numerical simulation analysis of the expansion fracture process of 45 steel cylindrical shell under different detonation pressure loading:

1. The experimental end-state fragmentation phenomenon of shear and tensile–shear mixed fracture under different explosive conditions can be simulated well by the SPH numerical model with a unified material constitutive and fracture model.
2. As the detonation pressure decreases, the fracture mode of 45 steel cylindrical shell changes from pure shear to tensile–shear mixed fracture. Cracks always form in the middle of shell thickness.
3. Under higher detonation pressure loading (JOB9003, RHT-901), the crack initiation and fracture of the 45 steel cylindrical shell occur during the loading stage; the failure of the cylindrical shell begins at mid-thickness in which the plastic strain accumulation is at its maximum. It spreads in the shear direction to the inner and outer walls, resulting in pure shear fracture mode.
4. As the detonation loading pressure decreases, loaded by explosives of TNT and HNS, on account of the initial fracture occurring during the loading stage, the fracture still begins from the middle of the shell thickness and spreads along the shear direction to the inner and outer walls. When the cylindrical shell expands to the free expansion stage, the unbroken area is under circumferential tensile stress, resulting in a phenomenon known as “necking”. The growing crack turns to the radial direction along the “necking” area, resulting in a tensile–shear mixed fracture mode. Furthermore, the lower the explosion pressure, the earlier the cylindrical shells enter the free expansion stage and the higher proportion of final tensile cracks.

## Figures and Tables

**Table 1:** Experimental cylindrical shell, loading conditions, and explosion–expansion fracture phenomenon, Refs. [15,33,34].

Types of Explosives	Wall Thickness/Inner Radius/d/R	δ_d_	$\varepsilon_{c}$	$t_{c}/\mu s$	$\varepsilon_{f}$	$t_{f}/\mu s$	$\dot{\varepsilon }/10^{4}\;s^{-1}$	Fracture Mode
RHT-901	5/30	0.17	0.18	7.8	0.37	15.8	2.5	tensile–shear mixed fracture
	4/30	0.13	0.24	8.8	0.43	15.4	2.9	tensile–shear mixed fracture
	3/30	0.10	0.30	10.1	0.45	14.1	3.5	pure shear
JOB9003	6/20	0.30	0.44	11.5	0.89	23.5	4.5	pure shear
	5/20	0.25	0.42	9.5	1.24	22.5	6.0	pure shear
	4/20	0.20	0.40	7.5	1.31	19.5	7.1	pure shear
	3/20	0.15	0.38	6.5	0.88	13.5	8.4	pure shear

Relative sized δ_d_ = shell thickness h/inner radius R of cylindrical shell $\varepsilon_{c}$—initial fracture strain, the radial strain at the moment $t_{c}$ when cracks appear on the outer wall of the cylindrical shell. $\varepsilon_{f}$—product leakage strain, the radial strain at the moment $t_{f}$ when the explosion products leak $\dot{\varepsilon }$—strain rate.

**Table 2:** Constitutive parameter values of 45 steel cylindrical shell [38].

Johnson–Cook						Grüneison State Equation		
A/MPa	B/MPa	n	m	C	$\dot{\varepsilon_{0}}$	C/m·s^−1^	s	$\gamma_{0}$
350	600	0.307	0.804	0.07	2 × 10^−4^	4600	1.49	2.17

**Table 3:** JWL EOS parameters of the explosives [39].

Types of Explosives	A/GPA	B/GPA	ω	$R_{1}$	$R_{2}$	$E_{0}$ $/GJm^{-3}$	$P_{cj}/\mathrm{GPA}$	ρ $/\mathrm{Kg}\cdot m^{-3}$	$D/m\cdot s^{-1}$
JOB9003	842.0	21.81	0.28	4.6	1.35	1	35	1884	8740
RHT-901	503.0	9.065	0.35	4.3	1.1	7.6	27	1658	7800

**Table 4:** Comparison of numerical simulation and experiment results.

Types of Explosives	R/h		$\varepsilon_{c}$	$t_{c}/\mu s$	$\varepsilon_{r}$	$t_{r}/\mu s$	$\varepsilon_{f}$	$t_{f}/\mu s$	$\dot{\varepsilon }/10^{4}\;s^{-1}$	Fracture Mode
RHT-901	30/3	experiment *	0.30	10.1	/	/	0.45	14.1	3.5	pure shear
		simulation	0.39	13.2	0.39	13.3	0.45	15.2	3.1	pure shear
	30/4	experiment *	0.24	8.8	/	/	0.43	15.4	2.9	tensile–shear mixed fracture
		simulation	0.30	13.1	0.31	13.5	0.41	16.9	2.7	tensile–shear mixed fracture
	30/5	experiment *	0.18	7.8	/	/	0.37	15.8	2.5	tensile–shear mixed fracture
		simulation	0.21	11.9	0.23	12.8	0.30	15.6	2.1	tensile–shear mixed fracture
JOB9003	20/3	experiment *	0.38	6.5	/	/	0.88	13.5	8.4	pure shear
		simulation	0.36	6.0	0.37	6.1	0.89	12.9	8.2	pure shear
	20/4	experiment *	0.40	7.5	/	/	1.31	19.5	7.1	pure shear
		simulation	0.37	7.3	0.37	7.3	1.07	16.7	7.1	pure shear
	20/5	experiment *	0.42	9.5	/	/	1.24	22.5	6.0	pure shear
		simulation	0.41	9.4	0.42	9.6	1.21	22.3	6.4	pure shear
	20/6	experiment *	0.44	11.5	/	/	0.89	23.5	4.5	pure shear
		simulation	0.43	11.4	0.45	11.7	0.90	20.0	5.4	pure shear

$\varepsilon_{c}$—initial fracture strain, the radial strain at the moment $t_{c}$ when cracks appear on the outer wall of the cylindrical shell; $\varepsilon_{f}$—products leakage strain, the radial strain at the moment $t_{f}$ when the explosion products leak $\dot{\varepsilon }$—strain rate; experiment *—data from the literature [30,31,32].

**Table 5:** Parameters related to the JWL constitutive equation of explosives.

Types of Explosives	A/MPA	B/MPA	ω	$R_{1}$	$R_{2}$	$E_{0}$ $/GJm^{-3}$	$P_{cj}/\mathrm{GPA}$	ρ $/Kg\cdot m^{-3}$	$D/m\cdot s^{-1}$
JOB9003	842.0	21.81	0.28	4.6	1.35	10	35	1884	8740
RHT-901	503.0	9.065	0.35	4.3	1.1	7.6	27	1658	7800
TNT	371.2	3.231	0.30	4.15	0.95	3	21	1730	6930
HNS	162.7	10.82	0.25	5.4	1.8	4.1	7.9	1000	5100

## Data Availability

Not applicable.
